# Fault analysis of chemical equipment based on an improved hybrid model

**DOI:** 10.1371/journal.pone.0326370

**Published:** 2025-07-18

**Authors:** Wu Huiyong, Kuan Jiang, Wang Yanyu

**Affiliations:** College of Science, Shenyang University of Chemical Technology, Shenyang, China; The Hong Kong Polytechnic University, CHINA

## Abstract

The safety and reliability of chemical equipment are crucial to industrial production, as they directly impact production efficiency, environmental protection, and personnel safety. However, traditional fault detection techniques often exhibit limitations when applied to the complex operational conditions, varying environmental factors, and multimodal data encountered in chemical equipment. These conventional methods typically rely on a single signal source or shallow feature extraction, which makes it difficult to effectively capture the deep, implicit information within the equipment’s operating state. Moreover, their accuracy and robustness are easily compromised when confronted with noisy signals or large, diverse datasets. Therefore, designing an intelligent fault detection method that integrates multimodal data, efficiently extracts deep features, and demonstrates strong generalization capability has become a key challenge in current research.This paper proposes an innovative fault detection method for chemical equipment aimed at improving detection accuracy and efficiency, providing technical support for intelligent and predictive maintenance. The method combines Variational Mode Decomposition (VMD), Least Mean Squares (LMS) processing, an asymmetric attention mechanism, and a pre-activation ResNet-BiGRU model to create an efficient framework for multimodal data fusion and analysis. First, the VMD-LMS process handles complex non-stationary signals, addressing the issue of mode mixing. Next, an asymmetric attention mechanism optimizes the ResNet, enhancing feature representation capabilities through deep learning. The pre-activation mechanism introduced in the residual blocks of ResNet improves training efficiency and model stability. Subsequently, the BiGRU model is used to model the extracted features in the time domain, capturing complex temporal dependencies. Experimental results demonstrate that the proposed method performs exceptionally well in chemical equipment fault detection, significantly enhancing diagnostic timeliness and reliability, achieving a classification accuracy of 99.78%, and providing an effective fault detection solution for industrial production.

## 1. Introduction

As the core component of mechanical transmission systems, bearings [1] play a critical role in supporting rotational motion and load-bearing in chemical equipment, directly influencing the equipment’s operational performance and safety. However, due to prolonged operation, load variations, as well as corrosion and contamination in the chemical environment, bearings are prone to various faults. These faults can lead to production line shutdowns, increased maintenance costs, and potentially serious safety incidents. Therefore, early detection and accurate diagnosis of bearing faults in the chemical industry have become pressing issues that need to be addressed. In recent years, data-driven intelligent diagnostic methods have gradually attracted widespread attention from researchers, providing new technological approaches to enhance the reliability and safety of chemical equipment.

In the field of bearing fault diagnosis, existing research methods can be categorized into three main types: model-driven methods [2], data-driven methods [3], and hybrid methods [4]. Model-driven methods rely on physical models to simulate the system’s working principles and fault mechanisms. These methods offer high interpretability and can provide scientific foundations. However, they are limited in terms of modeling accuracy and applicability when dealing with complex systems, multi-fault modes, or unknown external disturbances. Additionally, they often require domain expertise and can be costly. Data-driven methods, on the other hand, use large amounts of historical or real-time data, leveraging machine learning and deep learning techniques to construct diagnostic models. These methods automatically extract features and possess strong generalization and robustness, making them especially suitable for diagnosing high-dimensional, nonlinear, and complex fault modes. However, their “black-box” nature limits interpretability, and their performance may be compromised when data quality is low or sample sizes are insufficient. Hybrid methods combine the advantages of both model-driven and data-driven approaches, utilizing multimodal data fusion and model optimization to achieve higher diagnostic accuracy and adaptability. This has become a key direction in current research.

Currently, deep learning-based intelligent diagnostic methods have become mainstream, with technologies such as convolutional neural networks (CNN), bidirectional gated recurrent units (BiGRU), and attention mechanisms being widely applied in bearing fault diagnosis. For example, Zhao et al. [5] utilized a CNN-BiGRU Siamese network to enhance the model’s ability in spatial-temporal feature extraction, particularly excelling in capturing time-series features. Chen et al. [6] introduced a self-attention mechanism on top of CNN and BiGRU to improve feature extraction, particularly for signals with complex fault features. Zhang et al. [7] used preprocessed mechanical vibration signals to construct a BiGRU fault diagnosis model and applied attention mechanism-based model optimization to improve feature extraction efficiency. Wei et al. [8] proposed the use of Variational Mode Decomposition (VMD) on raw bearing vibration signals, followed by Hilbert-Huang Transform to address the “undershoot” issue in VMD. Jiang et al. [9] designed a feature extraction model based on bidirectional LSTM, combined with an adaptive attention mechanism and ResNet for feature learning, which allows for dynamic adjustment of the importance of key features. Liu et al. [10] proposed a bearing fault diagnosis method combining VMD optimized by a dung beetle algorithm and VGG (Visual Geometry Group) neural network, where the dung beetle algorithm effectively optimizes the VMD process. Chen et al. [11] proposed a bearing fault diagnosis method based on FFT and 1D-CNN, extracting frequency-domain features with FFT and automatically learning key features with 1D-CNN, achieving high computational efficiency, especially for periodic faults. Xie et al. [12] introduced a bearing fault diagnosis method that integrates channel attention mechanism, CNN, BiLSTM, and self-attention mechanism, optimizing CNN’s feature learning ability with channel attention, capturing multi-scale spatial features, modeling temporal information with BiLSTM, and focusing on critical fault features with the self-attention mechanism. Fan et al. [13] developed a method for high-speed rail bearing fault diagnosis by combining adaptive noise complete ensemble empirical mode decomposition with Fourier transform. They decomposed the signal and input the decomposed signals into BiLSTM for fault diagnosis. While these methods have improved fault diagnosis accuracy to some extent, there are still gaps in multimodal and multi-scale information fusion, especially in complex environments like chemical equipment. Existing methods still need further improvement in robustness and real-time performance.

To address the above issues, this paper proposes an improved bearing fault diagnosis method based on a modified residual network, integrating pre-activation and dual attention optimization. The model design fully considers the advantages and disadvantages of each module and the rationality of their combinations, forming a high-performance overall architecture. First, VMD is used to decompose the signal and extract modal features of different frequency components. The advantage of VMD is that it decomposes complex signals into multiple intrinsic mode functions (IMFs), enabling multi-scale feature analysis and solving the mode mixing problem encountered in traditional methods when dealing with non-stationary signals. However, VMD is sensitive to noise, and the decomposition results depend on parameter optimization. To address this, we introduce the LMS adaptive filtering algorithm to clean the decomposed signals. LMS can dynamically update filter weights in real-time, effectively suppressing noise and improving signal quality, thus compensating for VMD’s shortcomings in noise handling. This combination significantly enhances the clarity of the input signal, laying a foundation for subsequent feature extraction.

Next, the improved residual network is used for deep learning on the signal, extracting spatial patterns and key features. The improved pre-activation residual network introduces Batch Normalization (BN) and ReLU activation before convolution operations, mitigating the gradient vanishing problem in deep networks while accelerating training convergence. The pre-activation mechanism optimizes feature distribution by introducing non-linearity earlier, significantly improving network performance. However, traditional residual networks have limitations in feature importance perception. Therefore, we introduce an asymmetric attention mechanism, which optimizes feature weights through a combination of SE single-channel and SE dual-channel modules. Specifically, the SE single-channel module explicitly models each feature channel, capturing key features of individual modalities, while the SE dual-channel module considers the correlation and complementarity between features, enhancing the expression of multimodal features. The asymmetric attention mechanism dynamically adjusts the weights of feature channels based on these two modules’ characteristics, avoiding overly uniform attention weight allocation and focusing on the most relevant features.

In terms of the optimizer, the AdaBelief optimizer is employed. Compared to traditional optimizers, AdaBelief can dynamically adjust the learning rate based on the gradient’s variation, effectively avoiding overfitting, and complementing the efficient feature extraction of the residual network. To further capture temporal information in the signal, we introduce BiGRU for time-series modeling. The advantage of BiGRU lies in its bidirectional information flow design, allowing it to capture both forward and backward time-step information, making it superior to unidirectional GRU for modeling temporal dependencies. Additionally, the gating mechanism of BiGRU helps avoid the issues of gradient explosion and vanishing in long-term dependencies. However, BiGRU has higher computational complexity. Nevertheless, when combined with the aforementioned feature extraction modules, its precise modeling of temporal information significantly enhances the overall classification performance of the model, compensating for the limitations of static feature extraction methods in capturing temporal correlations.

Experimental results show that the proposed method achieves a classification accuracy of 99.78%, outperforming existing methods in terms of real-time performance, reliability, and fault classification accuracy, providing effective support for intelligent maintenance and predictive maintenance of chemical equipment. Furthermore, the design of this method addresses the challenges of complex operational conditions and multimodal data processing in chemical equipment, offering technical reference and theoretical foundations for fault diagnosis in complex industrial scenarios.

## 2. Theoretical basis and model construction

### 2.1. VMD (Variational Mode Decomposition)

VMD is a signal processing technique that decomposes a signal into a finite number of intrinsic mode functions (IMFs) or sub-signals using a variational approach. It extracts band-limited signals by defining the center frequency and bandwidth of each mode, and minimizes the error between the input signal and the sum of the modes through an optimization process. The key advantages of VMD include its ability to avoid mode mixing and effectively handle complex non-stationary signals. Additionally, VMD exhibits strong noise resistance, making it suitable for the analysis of various types of signals. The VMD decomposition process can be expressed as a constrained variational optimization problem, formulated as follows:


$$\underset{\left\{u_{k}\},\{\omega_{k}\right\}}{\min}\{\sum_{k}||\partial_{t}[(\delta (t)+\frac{j}{\pi t})u_{k}(t)]e^{-j\omega_{k}t}|{|}_{2}^{2}\}$$ (1)

$$s.t.\sum_{k}u_{k}=f$$ (2)

In the equation, *u*_*k*_ represents the *k* modal components obtained from the VMD decomposition; *ω*_*k*_ denotes the *k* central frequencies obtained from the VMD decomposition; “⋅” represents the convolution operation; In this context, *δ(t)* represents the Dirac delta function, *t* denotes the mathematical operation involved in gradient descent, and *f* is the input signal sequence subject to decomposition. By incorporating the penalty factor *α* and the Lagrange multiplier, the original constraint conditions are adjusted as illustrated in the following equation:


$$L=\alpha \sum_{k}||\partial_{t}[(\delta (t)+j\frac{1}{\pi t}{)}^{*}u_{k}(t)]e^{-j\omega_{k}t}|{|}_{2}^{2}+||f(t)-\sum_{k}u_{k}(t)|{|}_{2}^{2}+\left\langle \lambda (t),f(t)-\sum_{k=1}^{K}u_{k}(t)\right\rangle$$ (3)

Using the Alternating Direction Method of Multipliers(ADMM), the restricted variational equation’s optimal solution is found.The update formulas for the corresponding Fourier transforms are derived by solving for *u^*_*k*_, *ω^*_*k*_, and λ^ as follows:


$${\hat{u}}_{m}^{n+1}(\omega )=\frac{\hat{f}(\omega )-\sum {\mathrm{\backslash nolimits}}_{i\neq m}{\hat{u}}_{i}(\omega )+\frac{\hat{\lambda }(\omega )}{2}}{1+2\alpha (\omega -\omega_{m}^{2})}$$ (4)

$$\omega_{m}^{n+1}=\frac{\int_{0}^{\infty }\omega |{\hat{u}}_{m}^{n+1}(\omega ){|}^{2}d\omega }{\int_{0}^{\infty }|{\hat{u}}_{m}^{n+1}(\omega ){|}^{2}d\omega }$$ (5)

$$\hat{\lambda }n+1(\omega )={\hat{\lambda }}^{n}(\omega )+\gamma (\hat{f}(\omega )-\sum_{m}{\hat{u}}_{m}^{n+1}(\omega ))$$ (6)

In the equation: α is the penalty factor, which can reduce noise interference; γ is the noise tolerance; “ ” represents the Fourier transform. The iterative stopping criterion is:


$$\sum_{k=1}^{n}\frac{||{\hat{u}}_{m}^{n+1}(\omega )-{\hat{u}}_{k}^{n}(\omega )|{|}_{2}^{2}}{||{\hat{u}}_{k}^{n+1}(\omega )|{|}_{2}^{2}}<\varepsilon$$ (7)

In the equation,*ε*epsilon is the convergence threshold.

### 2.2. LMS (Least Mean Squares)

LMS algorithm is a classical adaptive filtering technique that iteratively updates the filter weights to minimize the mean squared error (MSE), thereby bringing the output signal as close as possible to the desired signal. The process begins by initializing the filter weights, typically set to a zero vector, w(0). Then, the input signal vector x(*n)* and the desired signal *d(n)*are fed into the system. Using the filter’s computation formula, the output *y(n)* is obtained, and the error *e(n)* is computed by comparing the desired signal *d(n)*with the output *y(n)*. The filter weights are then updated using the weight update formula, which gradually reduces the error. This process is repeated iteratively until the error converges to an acceptable range or the maximum number of iterations is reached. This iterative approach allows the filter to adapt in real time to changes in the input signal characteristics. The algorithm can be expressed as follows:

1. Error Calculation

e(n)=d(n)−y(n) (8)

*e(n)*: Error at the current time step

d*(n)*: Desired output at the current time step

*y(n)*: Actual output of the filter at the current time step

1. Filter output

y(n)=wT(n)x(n) (9)

*w(n)*: Filter weight vector

*x(n)*: Input signal vector

1. Weight update

w(n+1)=w(n)+2μe(n)x(n) (10)

μ: Learning rate, which controls the step size of weight updates

n: Number of iterations

### 2.3. Pre-activation residual network

The Pre-Activation Residual Neural Network (Pre-ResNet) is an enhancement of the traditional Residual Network (ResNet). The key distinction of Pre-ResNet lies in the positioning of the Batch Normalization (BN) and ReLU activation functions, which are placed before the convolutional layers within the residual block, rather than after as in the traditional post-activation structure. This adjustment leads to improved network optimization and more effective gradient propagation. The process is outlined as follows:

1. Batch Normalization: Normalize each sample using the calculated mean and variance, ensuring that the input has zero mean and unit variance.

$$B(x)=\frac{x_{i}-\mu_{B}}{\sqrt{{\sigma_{B}}^{2}+\varepsilon }}.\gamma +\beta$$ (11)

pre-activated convolutional layer:


$$H(x)=\gamma .\frac{x-\mu_{B}}{\sqrt{{\sigma_{B}}^{2}+\varepsilon }}+\beta$$ (12)

*H(x)* is the output after batch normalization, which can be adjusted using *β* and *γ.*

activation function:


A(x)=max(0,H(x)) (13)

residual connection and output:


$$y=W_{A}.A(x)+x$$ (14)

**Fig 1:**
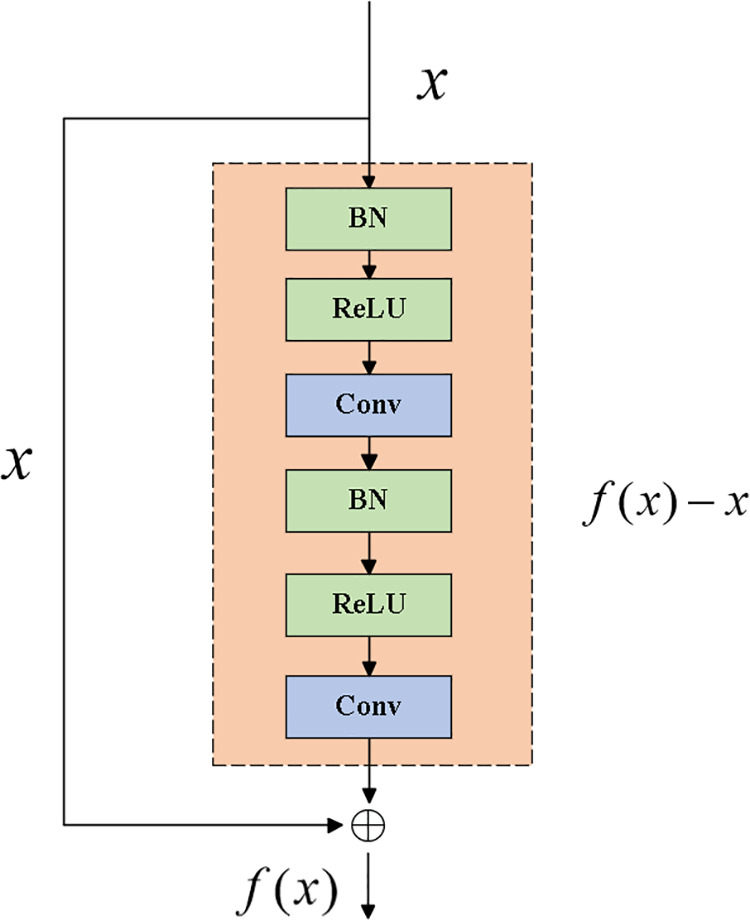
Pre-activation residual block diagram.

In this context, *W*_*A*_ represents the weights of the convolutional layer, *A(x)* is the output of the pre-activated convolutional layer, and *x* is the original input. By directly adding the output of the pre-activated convolutional layer to the original input, the gradient can flow more smoothly through the network, mitigating the issue of gradient vanishing during the training of deep networks. Unlike traditional residual blocks, the pre-activated residual block alters the order of the convolutional layer and activation function: batch normalization is applied first, followed by the ReLU activation function, and then the convolution operation. This adjustment not only accelerates the training process but also significantly improves the model’s convergence speed and stability, leading to superior performance when tackling complex tasks. The pre-activation residual structure is shown in Fig 1.

### 2.4. Attention mechanism

#### 2.4.1. SENet (Squeeze-and-Excitation Network).

SENet is a mechanism designed to enhance the performance of convolutional neural networks by explicitly modeling inter-channel dependencies. The core idea of SENet is to use the Squeeze-and-Excitation (SE) module to recalibrate the importance of each channel, thereby improving the network’s ability to selectively focus on relevant channel features. SENet introduces the SE block, which consists of three main steps: Squeeze, Excitation, and Recalibration.

The Squeeze process: Global average pooling reduces the spatial dimensions of each channel’s feature map into a single global feature, producing a vector whose length corresponds to the number of channels. This operation effectively transforms spatial information into global features that capture the overall semantics of each channel. The formula for this operation is as follows:


$$z_{c}=F_{sq}(x_{i})\frac{1}{H*W}\sum_{i=1}^{H}\sum_{j=1}^{W}X_{c}(i,j)$$ (15)

*Xc* is the *c*-th feature map, H*W represents the size of the feature map, and *Fsq(x*_*i*_) is the output value.

Excitation: The global feature vector is passed through a two-layer fully connected network (FC layers) with a bottleneck structure, first reducing and then expanding the number of channels. The intermediate layer typically employs the ReLU activation function, while the final layer uses the Sigmoid function to generate weights for each channel. These weights reflect the relative importance of each channel, with higher values indicating greater significance. The formula for this process is as follows:


$$s=F_{ex}[F_{sq}(x_{c}),W]=\sigma (W_{2}\delta \cdot (W_{1}F_{sq}(x_{c})))$$ (16)

Where *W*_*1*_ and *W*_*2*_ are the weight matrices of the two fully connected layers, *r* is the reduction ratio factor, and *σ* is the Sigmoid function.

Recalibration:The generated weights are applied to each channel of the original feature map (through channel-wise weighting), adjusting the features of each channel. This allows the network to focus more on the features of important channels. The formula is:


$${\tilde{X}}_{c}=s_{c}\cdot X_{c}$$ (17)

**Fig 2:**
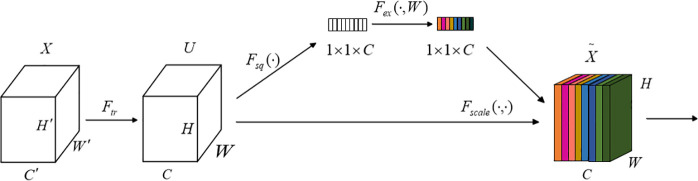
SENet attention mechanism.

**Fig 3:**
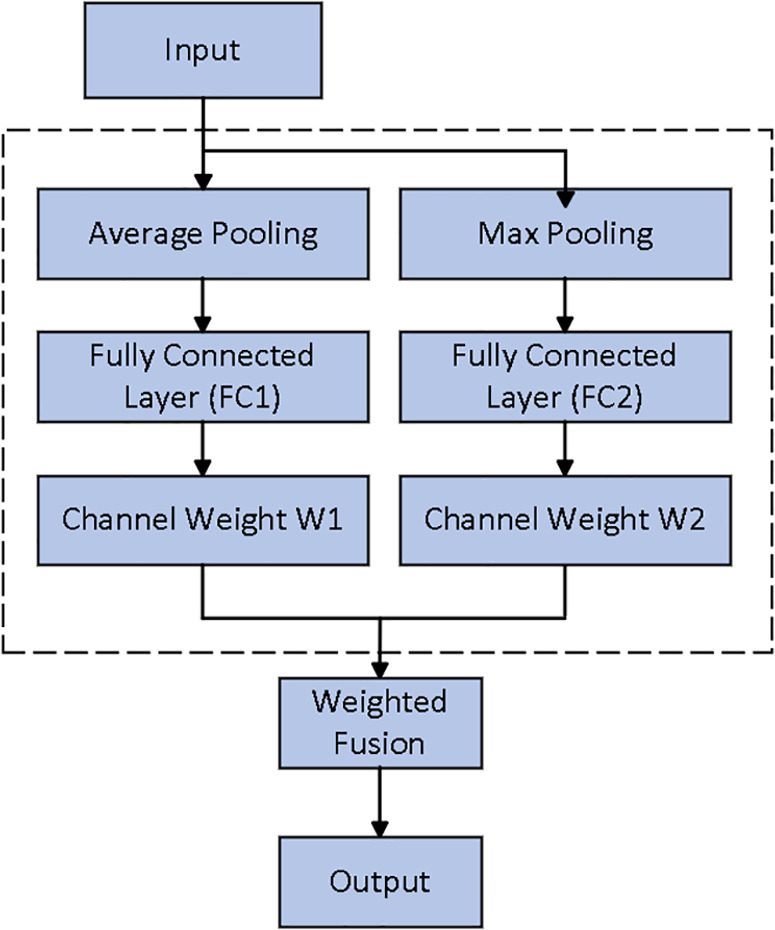
Dual-Channel SENet attention mechanism.

In deep learning, traditional attention mechanisms often rely on average pooling or max pooling to extract key information from the data. However, a single pooling method is typically insufficient for capturing the diversity and complexity of the data’s features. To address this limitation, this paper proposes an improved dual-channel attention mechanism, the Dual-Channel SE mechanism. This method divides the feature map channels into two groups, typically based on some inherent characteristics of the channels. Each group computes its own attention weights, which are then applied through separate excitation networks. This approach allows each group of channels to receive different weightings based on their distinct roles in the task. The weighted feature maps are then aggregated to produce the final output. The weighting proc ess enables the network to automatically focus on the more important channels while suppressing redundant or irrelevant ones. The Dual-Channel SE attention mechanism not only adjusts the importance of individual channels but also offers greater flexibility in tuning the weights of different features through the dual-channel strategy. The structure of the Dual-Channel SENet attention mechanism is shown in Figs 2 and 3.

#### 2.4.2. Asymmetric attention mechanism.

The asymmetric channel attention mechanism is a method designed to enhance the channel selectivity of feature maps. Unlike traditional channel attention mechanisms such as CBAM [14] and SE [15], the asymmetric channel attention mechanism applies different strategies and approaches to weight different channels, ensuring that each channel’s weight reflects its actual importance in the task at hand. By integrating both the single-channel and dual-channel SE attention mechanisms, the asymmetric channel attention mechanism further improves the channel selectivity of feature maps.

The single-channel SE attention mechanism extracts global information at the channel level through global average pooling and uses fully connected layers to generate the weights for each channel, thereby emphasizing important channel features. On the other hand, the dual-channel SE mechanism introduces additional channel information, enhancing the model’s ability to capture inter-channel dependencies and enabling more refined learning of the relationships between channels.

The network can flexibly apply different weighting strategies to various channel groups, ensuring that each channel’s attention weight aligns more closely with its importance in the task, thereby improving both feature extraction accuracy and classification performance.

**Fig 4:**
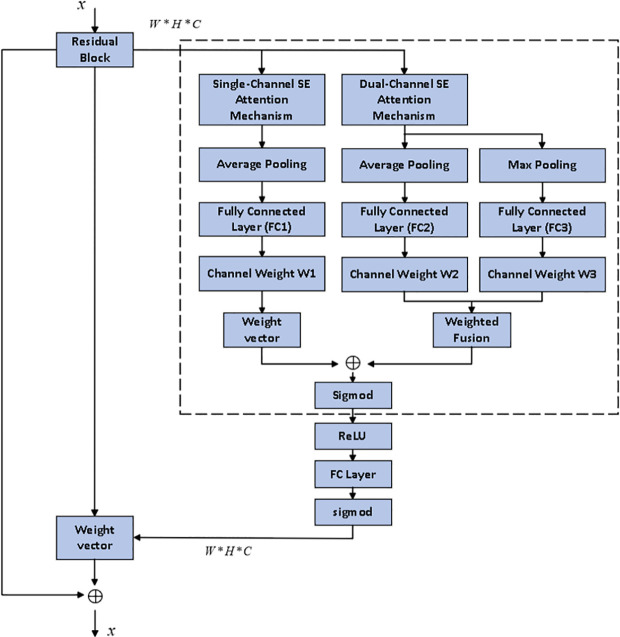
The residual network optimized by the asymmetric attention mechanism.

In the design of residual networks, the asymmetric channel attention mechanism is embedded within each residual block. The structure of the residual block allows the feature map to be directly added to the input after passing through the convolutional and activation layers, thereby mitigating the vanishing gradient problem. By incorporating the asymmetric channel attention mechanism into the residual blocks, the network can more precisely select and weight the channels of the feature map, enhancing both feature representation and the model’s classification performance. The diagram of the residual network structure optimized by the asymmetric attention mechanism is shown in Fig 4.

### 2.5. AdaBelief

AdaBelief [16] is an adaptive optimization algorithm that improves upon Adam by modifying the first moment estimate of gradients, enabling better adaptation to varying data distributions. Unlike Adam, which directly updates based on the gradient, AdaBelief focuses on the deviation between the gradient and its moving average. This allows for more adaptive adjustments to the learning rate. While retaining Adam’s fast convergence, AdaBelief enhances generalization and stability, effectively mitigating potential instabilities that can arise in certain tasks.

The algorithm starts by initializing parameters (*θ*_*0*_) and setting hyperparameters like the learning rate (*α*), momentum parameters (*β1, β2*), a small constant (*∊*) to avoid division by zero, and a bias correction parameter (*λ*). At each time step, it computes the current gradient (gt), updates the first moment (momentum), and estimates the second moment using the difference between the gradient and its first moment. Maximum second moment correction is applied to prevent excessive increases in the learning rate. Bias correction is applied to both moments before updating the parameters. These steps are repeated until the model converges or meets stopping criteria. The algorithm flow is shown in Table 1.

**Table 1:** The AdaBelief optimization process.

Step	Description
Initialize	$Set\alpha ,\beta_{1},\beta_{2},m_{0}=0,v_{0}=0,t=0$
Compute Gradient	$g_{t}=\nabla f\left(\theta_{t}\right)$
Update First Moment	$m_{t}=\beta_{1}m_{t-1}+\left(1-\beta_{1}\right)g_{t}$
Update Second Moment	$v_{t}=\beta_{2}v_{t-1}+\left(1-\beta_{2}\right){g_{t}}^{2}$
Compute Effective Moment	$s_{t}=\sqrt{v_{t}}+\varepsilon$
Update Parameters	$\theta_{t+1}=\theta_{t}-\frac{\alpha }{s_{t}}m_{t}$
Increment Time Step	t=t+1
Repeat	Repeat Steps 2–7 until convergence or max iterations.

### 2.6. BiGRU

The Bidirectional Gated Recurrent Unit (BiGRU) [17] is an advanced variant of the Recurrent Neural Network (RNN) that incorporates both bidirectional structure and gating mechanisms. This design enables BiGRU to capture both forward and backward dependencies in sequential data, significantly enhancing its ability to model complex sequences.

**Fig 5:**
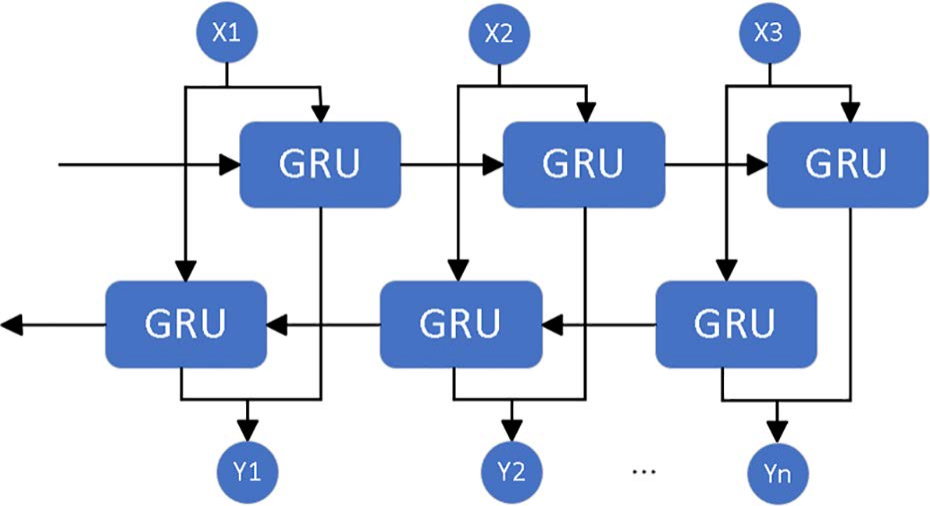
The structural diagram of BiGRU.

In a BiGRU, the sequence data is processed by two separate GRU units: one processes the sequence in the forward (positive) direction, while the other processes it in the backward (reverse) direction. The outputs from both directions are then concatenated or merged, resulting in a more comprehensive representation of the sequence. Its structure is shown in Fig 5.

#### 2.6.1. Update gate and reset gate.

The Gated Recurrent Unit (GRU) is a simplified variant of the Long Short-Term Memory (LSTM) network, utilizing gating mechanisms to regulate the updating and forgetting of information. The GRU is characterized by two primary gating mechanisms

This gate controls the amount of information from the previous time step that should be retained in the current hidden state. In the context of modeling equipment faults, especially in complex chemical systems, the update gate plays a crucial role in capturing long-term dependencies. Fault states in such equipment often exhibit significant historical dependencies, and the update gate enables the GRU to selectively retain important past information. This selective retention helps prevent issues like gradient vanishing or explosion, ensuring the model maintains both accuracy and stability over extended time periods. The mathematical formulation of the update gate is as follows:


$$z_{t}=\sigma (W_{z}\cdot [h_{t-1},x_{t}]+b_{z})$$ (18)

*σ* is the sigmoid activation function, *Wz* is the weight matrix for the update gate, *bz* is the bias term, *h*_*t−1*_ is the hidden state from the previous time step, and *x*_*t*_ is the input at the current time step.

Reset Gate: The reset gate controls how the current input and the previous hidden state information are combined. This mechanism enables the GRU to flexibly and selectively forget irrelevant historical information, retaining only the parts that are most pertinent to the current prediction task. By doing so, it allows the network to focus on the most relevant information, improving its ability to adapt to the dynamic nature of sequential data, such as fault signals in complex systems. The formula for the reset gate is as follows:


$$r_{t}=\sigma (W_{r}\cdot [h_{t-1},x_{t}]+b_{r})$$ (19)

*r*_*t*_ is the reset gate’s output at time step *t.σ* is the sigmoid activation function, with an output range of 0–1. *Wr* refers to the reset gate’s weight matrix. *h*_*t−1*_ represents the concealed state from the previous time step. *x*_*t*_ represents the input at the current time step. *br* represents the reset gate’s bias.

#### 2.6.2. Candidate hidden state.

The candidate hidden state combines the current input and the information controlled by the reset gate, representing the potential state at the current time step. This mechanism allows the GRU to effectively capture local temporal features and extract key information in subsequent computations, thereby providing more valuable features for future time-series modeling. The formula is:


$$h_{t}=\text{tanh}(W_{h}\cdot [r_{t}⊙h_{t-1},x_{t}]+b_{h})$$ (20)

Here, *W*_*h*_ is the weight matrix for the candidate hidden state, *b*_*h*_ is the bias term

#### 2.6.3. Final hidden state.

The final hidden state is the weighted average of the current hidden state (controlled by the update gate) and the candidate hidden state, combining historical information and current input features. This weighting mechanism allows the GRU to dynamically adjust the weight between historical information and current input, further enhancing its ability to extract fault features from equipment. The formula is:


$$h_{t}=(1-z_{t})⊙h_{t-1}+z_{t}⊙{\tilde{h}}_{t}$$ (21)

## 3. Bearing fault simulation and experimental results analysis

### 3.1. Improved ResNet-BiGRU neural network

The proposed improved ResNet-BiGRU method begins by applying pre-activation to the residual module, adjusting the Batch Normalization (BN) layer and ReLU activation function to precede the convolutional layer. Subsequently, the asymmetric channel attention mechanism is embedded within each residual block. This structure allows the feature map, after passing through convolution and activation layers, to be directly added to the input in the ResNet residual network. The features are then weighted and adjusted to effectively capture multi-scale features and optimize feature weights.

In terms of hyperparameter optimization, AdaBelief is used to replace the traditional Adam optimizer. Finally, a BiGRU is introduced to extend the time-domain feature capture capabilities of the ResNet. BiGRU, through forward and backward gated recurrent units, effectively captures long-term dependencies in sequence data, uncovering the inherent patterns in the time-series information of equipment operating conditions. Its bidirectional information flow design enables the model to consider both historical data and future data, thereby enhancing its understanding of temporal information and improving fault diagnosis accuracy and robustness.

**Fig 6:**
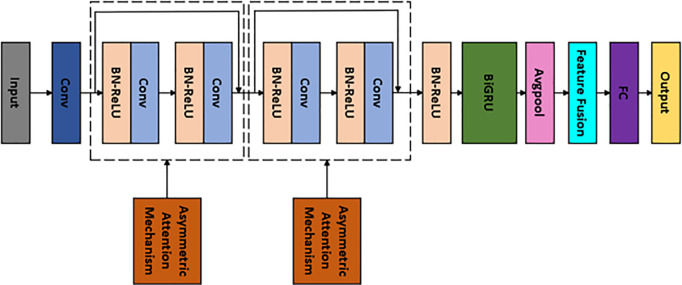
Improved ResNet-BiGRU Model.

**Fig 7:**
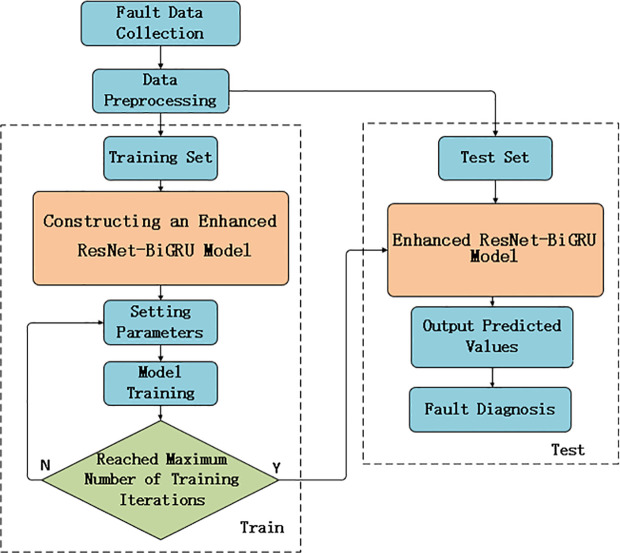
The fault diagnosis process based on the improved ResNet-BiGRU model.

**Fig 8:**
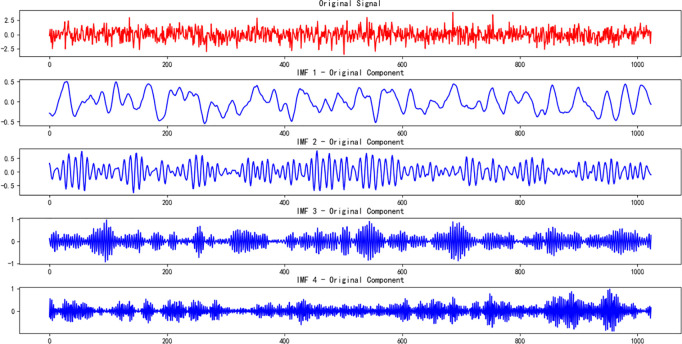
Visualization of fault VMD decomposition.

Overall, the improved ResNet-BiGRU model achieves efficient feature extraction and fault diagnosis classification of multi-dimensional process data, demonstrating excellent performance and generalization ability. The overall structure of the model and the fault diagnosis process are shown in Figs 6–8, respectively, with detailed parameters provided in Table 2.

**Table 2:** The AdaBelief optimization process.

Layer name	Layer type	Filter	Stride
Input	Input	–	–
Conv1	Conv1	3 × 3,64	1 × 1
ResNet1	BN-ReLU	–	–
	Conv1	3 × 3,128	1 × 1
	BN-ReLU	–	–
	Conv1	3 × 3,128	1 × 1
Asymmetric Attention Mechanism	–	–	–
ResNet2	BN-ReLU	–	–
	Conv1	3 × 3,256	1 × 2
	BN-ReLU	–	–
	Conv1	3 × 3,256	1 × 1
Asymmetric Attention Mechanism	–	–	–
BiGRU	–	2,128	–
Avgpool	AVgpool	–	–
FC	FC	10	–

### 3.2. Case study

#### 3.2.1. Data source.

The CWRU bearing dataset is a globally recognized standard for bearing fault diagnosis. Consists of a motor, torque sensor/encoder, dynamometer, and control electronics. The bearings used for testing are SKF6205 motor bearings, with motor loads of 0HP, 1HP, 2HP, and 3HP, and sampling frequencies of 12 kHz and 48 kHz. In this study, the data from the drive-end accelerometer, sampled at 12 kHz and with a load of 2HP, was used for experimentation. The bearing faults were introduced using electrical discharge machining, including inner race faults, outer race faults, and ball faults, with fault diameters of 0.1778 mm, 0.3556 mm, and 0.5334 mm, respectively. Based on fault location and diameter, the dataset is categorized into ten classes: Normal (Normal), Inner Race (IR007, IR014, IR021), Outer Race (OR007, OR014, OR021), and Ball Fault (B007, B014, B021). A total of 9,350 samples were used, with the dataset split into training, validation, and testing sets in a 7:1:2 ratio. The specific parameters of the dataset are shown in Table 3.

**Table 3:** The parameters of the CWRU bearing dataset.

Location	Diameter	Category	Label
Inner Race	0.1778	IR007	1
	0.3556	IR014	2
	0.5334	IR021	3
Outer Race	0.1778	OR007	4
	0.3556	OR014	5
	0.5334	OR021	6
Ball Bearing	0.1778	B007	7
	0.3556	B014	8
	0.5334	B021	9
normal	0	Normal	10

Additionally, the MFPT bearing fault dataset was used in the experiments, which includes ten categories: normal rolling bearing data (NO), intermediate shaft bearing data from a wind turbine under real operating conditions (ISB), inner race fault data (IR25, IR150, IR300, with loads of 25, 150, and 300 pounds, respectively), oil pump shaft bearing data from a wind turbine under real operating conditions (OPB), outer race fault data (OR0, OR150, OR300, with loads of 0, 150, and 300 pounds, respectively), and planetary bearing fault data (PB). The normal data was sampled at 97,656 samples per second (sps), while the inner race and outer race fault data were sampled at 48,828 sps. Each category contains 3–6 seconds of signal data, and 1,000 samples were selected from each category for analysis. Each sample has a length of 1,200 data points. The dataset was divided into training and testing sets, with the specific sample names and sizes listed in Table 4.

**Table 4:** The parameters of the MFPT bearing dataset.

Type	Train	Test	Label
NO	800	200	1
ISB	800	200	2
IR25	800	200	3
IR150	800	200	4
IR300	800	200	5
OPB	800	200	6
OR0	800	200	7
OR150	800	200	8
OR300	800	200	9
PB	800	200	10

#### 3.2.2. VMD feature extraction.

**Fig 9:**
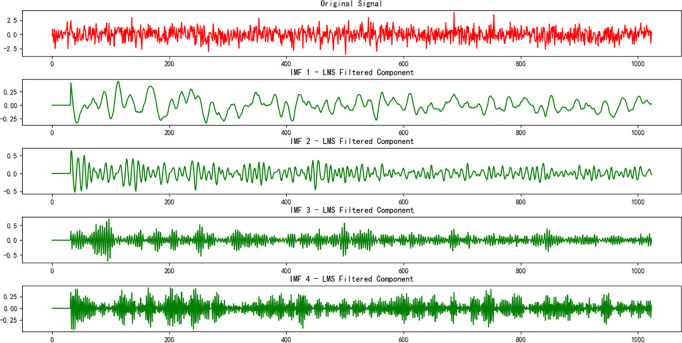
Visualization of fault LMS decomposition.

VMD [18] is an adaptive modal decomposition method that decomposes a signal into multiple intrinsic mode functions (IMFs), reflecting the signal’s features at different scales. By using VMD, the multi-scale time-domain features of the signal can be extracted, providing a better understanding of its time-frequency characteristics. In this study, the VMD-decomposed IMFs are further processed with the LMS (Least Mean Squares) algorithm for filtering, which reduces noise and enhances the signal’s features. After fusing the features extracted by VMD and LMS, they are fed into an innovative network model for training and optimization. The visualization of the fault VMD decomposition and LMS algorithm is shown in Figs 7–9, with the relevant parameters provided in Table 5.

**Table 5:** Translation of detailed parameters of VMD decomposition and LMS model.

Model	R/%	R/%
VMD	alpha	2000
	tau	0
	K	5
	DC	0
	init	1
	tol	1e − 7
LMS	mu	0.01
	N	2000

#### 3.2.3. Model trainin.

**Fig 10:**
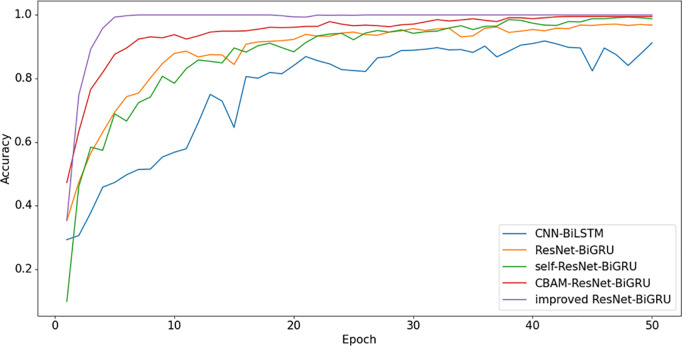
Comparison of accuracy curves for five models in the CWRU dataset.

**Fig 11:**
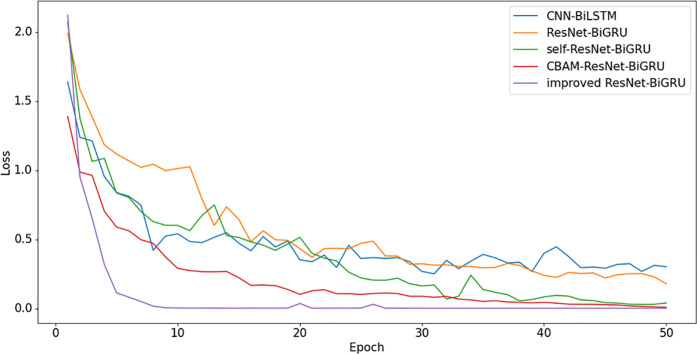
Comparison of loss curves for five models in the CWRU dataset.

To fully demonstrate the performance advantages of the proposed improved ResNet-BiGRU model, this study compares it with several other models, including CNN-BiLSTM [19] and the original ResNet-BiGRU model. To highlight the effectiveness of the attention mechanisms, Self-Attention and CBAM-Attention are incorporated into the ResNet [20]-BiGRU model, and comparisons are made with the approach presented in this study. By comparing the Accuracy and Loss curves of different models, as shown in Figs 10 and 11, performance differences across the models can be observed. Statistical data analysis reveals that the proposed improved ResNet-BiGRU model achieves the best average accuracy. The Loss curve indicates that as the number of training iterations increases, the model’s loss gradually decreases and eventually stabilizes, reaching convergence after approximately 4 iterations. Meanwhile, the Accuracy curve shows a steady upward trend, reaching an accuracy of 99.78% on the test set, which is an improvement of 2.45% compared to the original ResNet-BiGRU model. Moreover, when compared to other models, the improved model outperforms them all, significantly enhancing overall accuracy. This result demonstrates that the improved model not only achieves high fault diagnosis accuracy but also avoids overfitting, making it a more robust solution for fault detection. Its high accuracy and stability make it an efficient neural network architecture for fault diagnosis. The Final Training Results of each model is shown in Table 6.

**Table 6:** Final training results.

Dataset	Model	ACC/%	R/%	P/%	F1-score/%
**CWRU**	CNN-BiLSTM	95.71	95.52	93.65	95.52
	ResNet-BiGRU	97.33	97.33	96.15	97.35
	Self-ResNet-BiGRU	98.62	98.61	98.97	98.65
	CBAM-ResNet-BiGRU	99.68	99.62	99.29	99.66
	Improve-ResNet-BiGRU	99.78	99.91	99.82	99.94
**MFPT**	CNN-BiLSTM	95.31	95.22	94.58	95.66
	ResNet-BiGRU	96.89	97.01	97.15	97.33
	Self-ResNet-BiGRU	97.91	98.05	98.08	98.33
	CBAM-ResNet-BiGRU	99.02	99.35	99.11	99.07
	Improve-ResNet-BiGRU	99.78	99.88	99.72	99.78

To further evaluate the performance of different models in fault diagnosis, this study conducted a quantitative analysis using multiclass confusion matrices for the CNN-BiLSTM model, the original ResNet-BiGRU model, the CBAM-ResNet-BiGRU model, and the proposed improved ResNet-BiGRU model. As an intuitive evaluation tool, confusion matrices clearly reveal each model’s accuracy in identifying and classifying various fault categories. In these matrices, columns represent the actual fault categories of the samples, while rows denote the predicted categories by the models. The diagonal values indicate the proportion of correctly classified samples, and the rightmost column of the matrix provides a straightforward representation of each model’s overall classification accuracy.4

**Fig 12:**
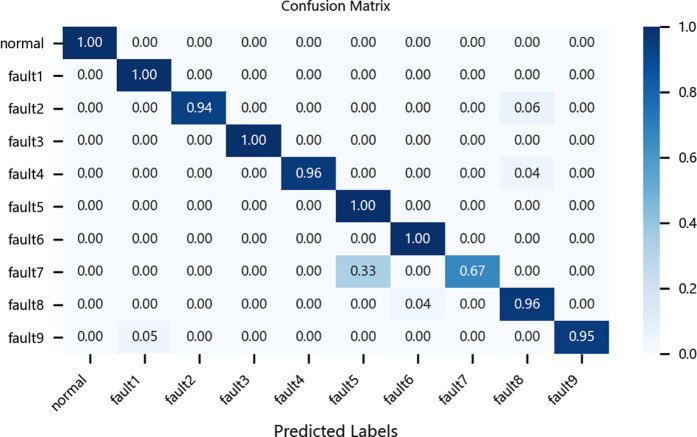
Confusion matrix for CNN-BILSTM.

**Fig 13:**
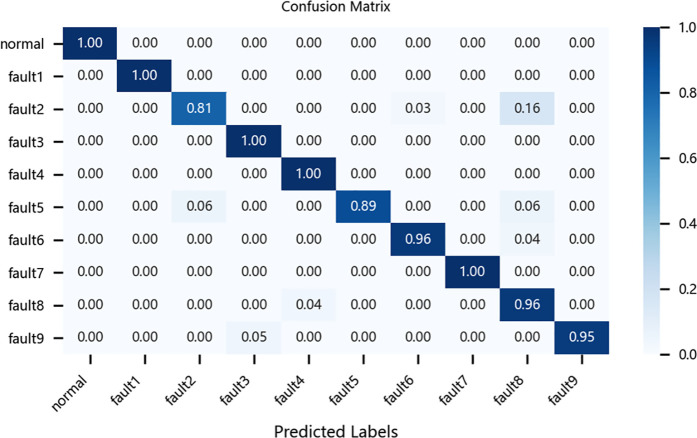
Confusion matrix for ResNet-BIGRU.

**Fig 14:**
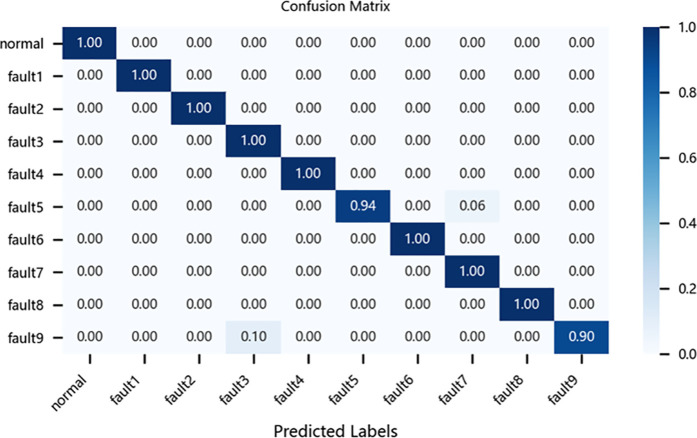
Confusion matrix for CBAM-ResNet-BiGRU.

**Fig 15:**
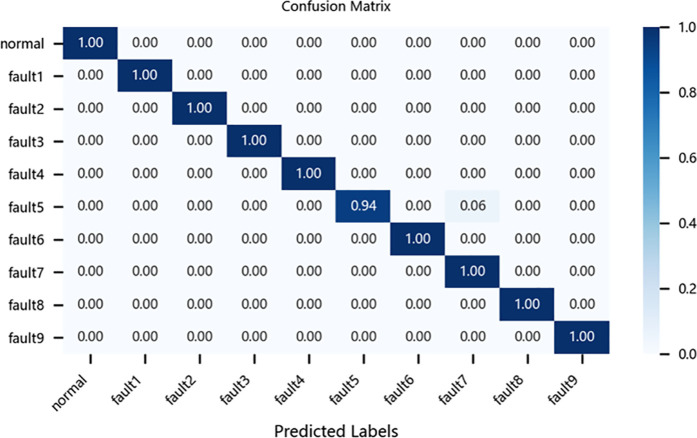
Confusion matrix for Improve-ResNet-BiGRU.

Taking the CWRU dataset as an example, the relevant confusion matrices are shown in Figs 12–15. The analysis of these matrices reveals that the improved ResNet-BiGRU model achieves a 100% diagnosis rate for 9 out of 10 fault categories, with only Fault 5 having a diagnosis rate of 94%. This demonstrates superior overall diagnostic performance. In contrast, the CNN-BiLSTM model achieves a 100% diagnosis rate for only 5 fault categories and performs significantly worse in diagnosing Faults 2 and 8 compared to the improved model. The original ResNet-BiGRU model shows substantial improvement overall, while the CBAM-ResNet-BiGRU model achieves a 100% diagnosis rate for 8 fault categories but is slightly less effective in diagnosing Faults 5 and 10 compared to the improved ResNet-BiGRU model.

The enhancements introduced through the residual structure have significantly improved fault diagnosis rates. Comparative results indicate that the improved ResNet-BiGRU model exhibits a distinct advantage over other models in accurately distinguishing single faults, demonstrating its superior diagnostic capability.

#### 3.2.4. Data visualization.

To further investigate the structure and classification process of the improved ResNet-BiGRU model, t-SNE [21] (t-distributed Stochastic Neighbor Embedding) was employed for the visualization analysis of the sample test data. In this study, 1,870 samples from the fault dataset were selected as the test set. The t-SNE method was then applied to map the high-dimensional data into a two-dimensional space, enabling a better observation of the data clustering.

**Fig 16:**
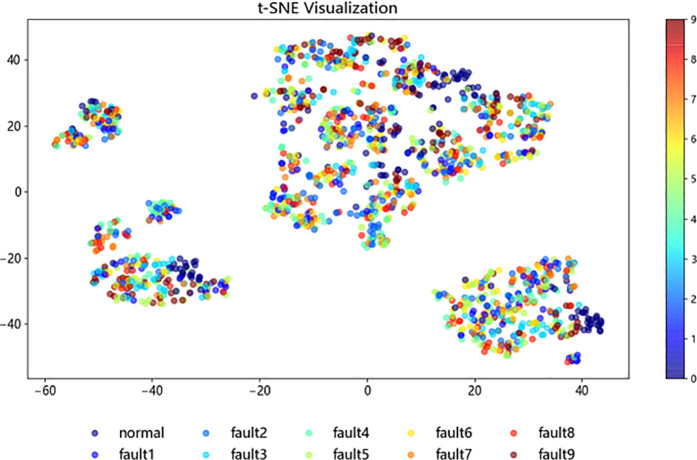
Intput layer.

**Fig 17:**
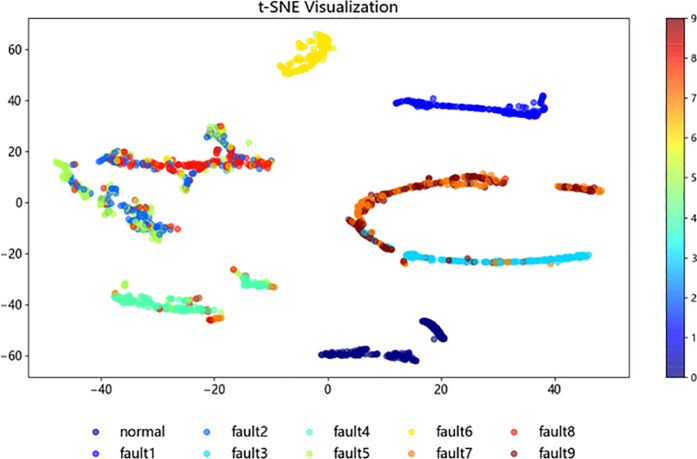
ResNet layer.

**Fig 18:**
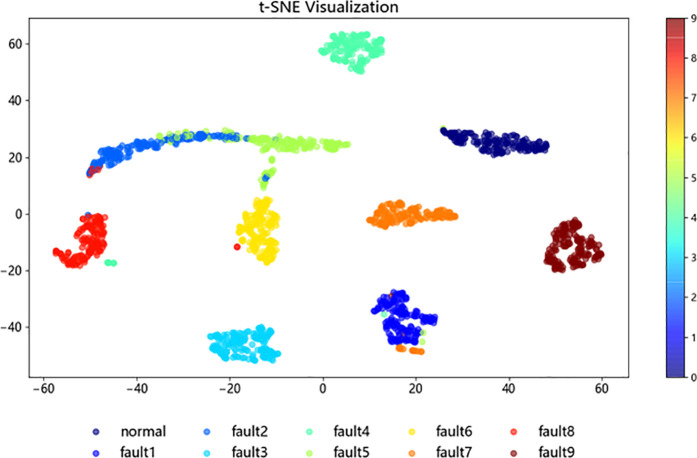
BIGRU layer.

**Fig 19:**
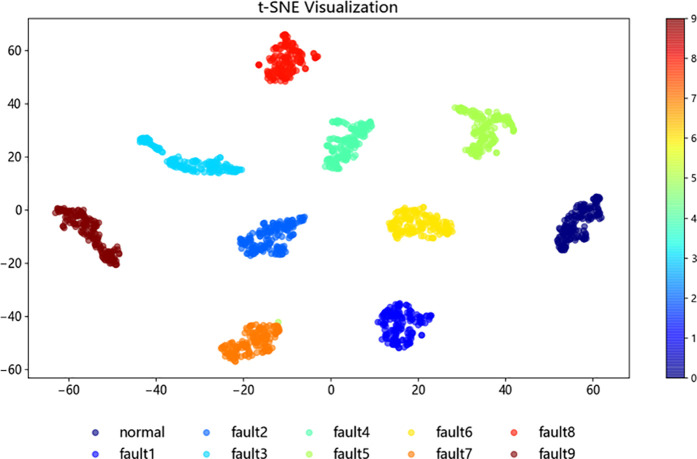
Output layer.

Figs 15–18 illustrate the learning process of the training data in t-SNE. By projecting the 10 different fault categories into a two-dimensional space and using distinct colors to represent each category, each point corresponds to a sample. From Fig 15, it is observed that the original samples are mixed together, showing poor classification performance and difficulty in distinguishing between categories. In Fig 16, following the feature extraction in the improved ResNet [22] layer, most faults are clearly separated in the two-dimensional space, indicating that the ResNet-BiGRU model effectively extracts key features. Fig 17 then shows the data distribution after entering the BiGRU layer for temporal feature extraction, where only a small number of hard-to-diagnose faults are misclassified. Finally, Fig 18 presents the model’s final output, where the clustering effect is more prominent after processing through the entire model, further demonstrating the model’s superior fault diagnosis capability Fig 19.

## 4. Conclusion

The proposed improved ResNet-BiGRU model integrates several advanced techniques for bearing fault analysis. First, the VMD algorithm is used to decompose the spectral signal and extract time-frequency features, which are then enhanced using the LMS algorithm to improve signal quality. Next, the pre-activated ResNet network, optimized with an asymmetric channel attention mechanism, adjusts the weights of the features and captures multi-scale characteristics. The AdaBelief optimizer is employed in place of the traditional Adam algorithm. Finally, the BiGRU model is introduced to strengthen the ability to capture temporal features. By leveraging bidirectional information flow, BiGRU effectively mines the long-term dependencies in the sequential data, enhancing both the accuracy and robustness of fault diagnosis. Overall, the improved ResNet-BiGRU model achieves efficient feature extraction and fault diagnosis, demonstrating excellent performance and generalization capabilities.

## Supporting information

S1 File Data_12k_10c. (CSV)
